# BrainSeg-Net: Brain Tumor MR Image Segmentation via Enhanced Encoder–Decoder Network

**DOI:** 10.3390/diagnostics11020169

**Published:** 2021-01-25

**Authors:** Mobeen Ur Rehman, SeungBin Cho, Jeehong Kim, Kil To Chong

**Affiliations:** 1 Department of Electronics and Information Engineering, Jeonbuk National University, Jeonju 54896, Korea; cmobeenrahman@jbnu.ac.kr (M.U.R.); seunbgin1647@jbnu.ac.kr (S.C.); 2 Department of Avionics Engineering, Air University, Islamabad 44000, Pakistan; 3 Department of New & Renewable Energy, VISION College of Jeonju, Jeonju 55069, Korea; 4 Advanced Electronics and Information Research Center, Jeonbuk National University, Jeonju 54896, Korea

**Keywords:** medical imaging, semantic segmentation, brain tumor, diagnostics, Feature Enhancer (FE), Magnetic Resonance (MR) Images

## Abstract

Efficient segmentation of Magnetic Resonance (MR) brain tumor images is of the utmost value for the diagnosis of tumor region. In recent years, advancement in the field of neural networks has been used to refine the segmentation performance of brain tumor sub-regions. The brain tumor segmentation has proven to be a complicated task even for neural networks, due to the small-scale tumor regions. These small-scale tumor regions are unable to be identified, the reason being their tiny size and the huge difference between area occupancy by different tumor classes. In previous state-of-the-art neural network models, the biggest problem was that the location information along with spatial details gets lost in deeper layers. To address these problems, we have proposed an encoder–decoder based model named BrainSeg-Net. The Feature Enhancer (FE) block is incorporated into the BrainSeg-Net architecture which extracts the middle-level features from low-level features from the shallow layers and shares them with the dense layers. This feature aggregation helps to achieve better performance of tumor identification. To address the problem associated with imbalance class, we have used a custom-designed loss function. For evaluation of BrainSeg-Net architecture, three benchmark datasets are utilized: BraTS2017, BraTS 2018, and BraTS 2019. Segmentation of Enhancing Core (EC), Whole Tumor (WT), and Tumor Core (TC) is carried out. The proposed architecture have exhibited good improvement when compared with existing baseline and state-of-the-art techniques. The MR brain tumor segmentation by BrainSeg-Net uses enhanced location and spatial features, which performs better than the existing plethora of brain MR image segmentation approaches.

## 1. Introduction

In modern society, diseases related to the brain are emerging as a big problem especially malignant brain tumors which are greatly influencing human lives [1]. Gliomas are the most-occurring malignant brain tumor, they are caused by abnormal cell transformation, and are largely classified into High-Grade Gliomas (HGG) and Low-Grade Gliomas (LGG) [2]. HGG are malignant tumors that have already grown; their progress has considerably deteriorated and surgery is essential. LGG is less advanced than HGG, and life expectancy can be extended through treatment [3]. There are different methods to distinguish these tumor lesions: Computed Tomography (CT), X-ray, Single-Photon Emission Computed Tomography (SPECT), Ultrasound, Magnetic Resonance Imaging (MRI), Positron Emission Tomography (PET), Magnetic Brain Wave Graph (MEG), and Electroencephalogram (EEG) [4]. However, among all medical imaging techniques, MRI is considered to be the most comprehensive method which can help to to determine the exact size and volume of the malignant tumor [5]. The images generated by MRI are used to measure and analyze the location and size of the tumor, and can be divided according to the characteristics of the tumor, which can be improved with an optimal diagnostic process and treatment method. Because of the high quality of MRI, effective segmentation of brain tumors has become one of the most important research problems in the field of medical imaging [6].

MRI segments a brain tumor through four visualized images with different characteristics. These imaging methods areT1-weighted (T1w), T1w contrast-enhanced (CE), T2-weighted (T2w), and Fluid-Attenuated Inversion Recovery (FLAIR). The T1 image is used to differentiate healthy tissue, and T2 is used to describe the edema area that produces a bright signal. The T1ce can be distinguished by a bright signal from the contrast agent, which has accumulated in the tumor boundary and the active cell area of the tumor tissue. Flair differentiates between edema and cerebrospinal fluid by inhibiting the signals of water molecules [1]. Based on this, tumors can be subdivided into tumor nuclei, reinforced tumors, and whole tumors. Some tumors, such as meningiomas, segment easily, but gliomas and glioblastoma cells spread well and are difficult to segment because these are not contrasted [7]. Besides, it is not easy to segment because it can occur at any location and varies in size.

The brain tumor segmentation models are generally divided into 2D data and 3D data-based models. Several researches [8,9,10,11] have demonstrated that 3D architectures perform better than 2D architectures. However, 3D architectures have limitations as they use more parameters and are computational complex [12]. Specifically, the dataset utilized for applying the 3D model is often reduced to half the size of the existing training data.

In this research, we put forward a model named BrainSeg-Net. BrainSeg-Net uses a FE block, which is added to enhance the features of shallow layers before adding them to deep layers, contributing towards the classification and segmentation process. Moreover, the feature maps extracted by FE block are upsampled and concatenated with the result of the higher-level encoder–decoder connection. This increases the valid receptive field of the network which helps to locate the small-scale tumors.

The remaining paper is structured as follows. Section 2 discusses the related work, Section 3 gives the details regarding datasets used to evaluate the model, Section 4 explains the proposed model, Section 5 presents the results achieved by BrainSeg-Net, and Section 6 concludes the paper.

## 2. Related Work

In the past few years, various deep learning models for computer vision tasks have been proposed, such as VGGNet [13], ResNet [14], and DenseNet [15]. Deep Neural Networks have the strong ability to automatically extract the discriminant features; therefore, they are widely used in the field of medical imaging and bioinformatics [16,17,18,19,20]. Similarly, the use of deep learning for computer-aided diagnosis of brain tumor has gathered extensive attention. In recent years, Medical Image Computing and Computer-Assisted Invention (MICCAI) and Brain Tumor Segmentation (BraTS) challenge have greatly contributed towards the development of neural network-based architectures for brain tumor diagnosis.

Broadly, the methods for image segmentation based on deep learning can be of two types which are Convolutional Neural Network (CNN) and Full Convolution Network (FCN). CNN based methods use small patch classification technique for tumor segmentation. Havaei et al. applied a segmentation method using CNN architecture to segment brain tumor regions from 2D MRI images and used convolutional kernels of various sizes to extract important contextual features [7]. Another CNN-based technique was proposed by Pereira et al. which is an automated segmentation architecture based on VGG-Net. However, the CNN architecture is a patch-based method, which requires large storage space and lacks spatial continuity, resulting in poor efficiency. On other hand, FCN techniques perform calculation pixel by pixel based on the Encoder–Decoder concept, this not only increases the brain tumor segmentation efficiency but also the spatial continuity problem gets solved [21].

Based on the FCN concept, Ronneberger et al. [22] proposed a U-Net architecture applied to various medical segmentation problems. U-Net is based on the conception of encoding and decoding paths, where encoding path extracts the contextual features while decoding path keeps an accurate track of the location. The concatenation of encoder and decoder features in U-Net architecture greatly improves the medical image segmentation performance. U-Net architecture is widely used by researchers for brain tumor segmentation. Dong et al. proposed a 2D segmentation network based on U-Net along with which they performed real-time data augmentation to increase the performance of brain tumor segmentation [23]. Kong et al. added the feature pyramid module in U-Net architecture to increase the accuracy by integrating location details and multi-scale semantic [24]. Extensive work has been carried out to improve the performance of U-Net for image segmentation like using dilated convolution, introducing up skip connection, using dense block and embedding MultiRes block in base U-Net architecture [25,26,27,28]. For further improvement in U-Net architecture, the Attention Gates (AGs) were integrated into the architecture [29]. The addition of Squeeze-and-Excitation (SE) blocks [30] was made in [31,32] for prostate zonal segmentation on MR images. Vasileios et al. propose a skull stripping technique which classifies skull-free images to give better segmentation performance. McKinley et al. developed a network by applying the extended convolution structure [33]. Wang et al. proved that the brain segmentation performance improves when convolution neural networks are applied with Test Time Augmentation technique (TTA) [34].

Undoubtedly, U-Net has shown great success in the medical field and therefore presently it is mainstream of segmentation architectures for brain tumor MRI. However, the encoder side of the U-Net architecture performs downsampling which reduces the image size, resulting in low performance as small-scale tumors are unable to be identified. To solve this problem, a mechanism needs to be adopted that enhances the local features and help the decoder to locate the tumor efficiently.

## 3. Datasets

**Figure 1:**
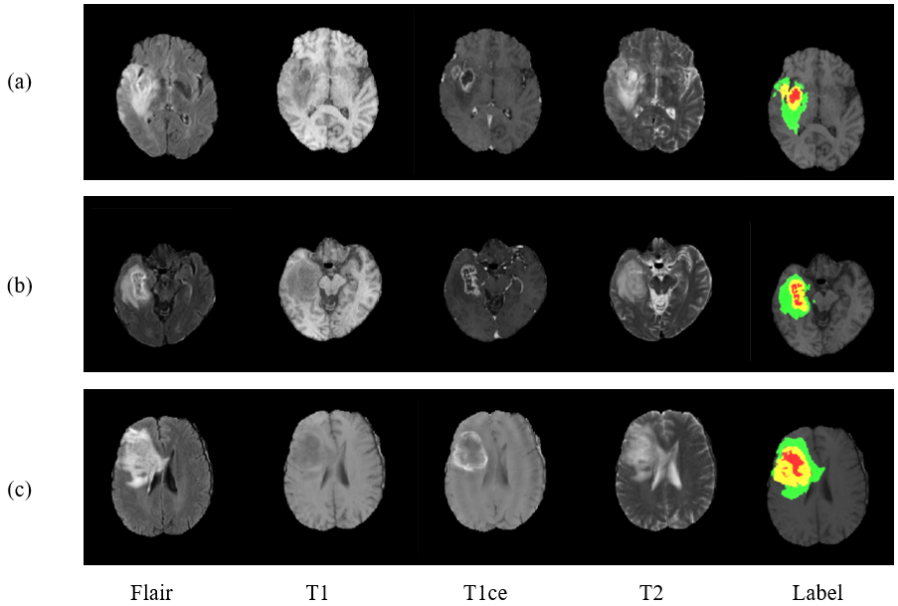
Presenting three MRI cases (**a**–**c**) of brain tumor with four modalities and a label plots. From left to right are Flair, T1, T1ce, T2, and Label. In ground truth image each color represents different tumor class. Red for necrosis and non-enhancing, green for edema, and yellow for enhancing tumor.

For the evaluation of proposed model, three benchmark databases are taken into consideration: BraTS 2017, Brats 2018, and BraTS 2019. These are publicly available benchmark databases used to train and evaluate the model. The BraTS2017 dataset is a collection of data from 285 glioma patients, consisting of 210 HGG and 75 LGG cases. The validation data set additionally includes images of 46 patients having an unknown grade. Unlike training data, validation data are not labeled, and results can only be generated using the online web of BraTS challenge. The dataset includes all four modalities for every patient as can be seen in Figure 1, where three data of three different patients is exhibited.

There are four labels in the dataset:

- Necrosis and Non-enhancing Tumor
- Enhancing Tumor
- Healthy Tissue
- Edema

The training dataset of BraTS 2018 is similar to BraTS2017 but validation dataset is different. The validation dataset of BraTS 2018 has more cases than BraTS 2017 which are 66. There is a different training dataset of BraTS 2019 which have more number of cases than previous databases. BraTS 2019 comprises of 335 glioma cases, where 259 belongs to HGG and remaining 76 belongs to LGG. Further, BraTS 2019 has an expanded validation dataset which carried 125 cases.

## 4. Methodology

This section discusses the preprocessing process carried out on the input images along with architecture details of BrainSeg-Net. Further, the BrainSeg-Net also discusses the FE block and custom-designed loss function.

### 4.1. Image Preprocessing

Deep Learning models are undoubtedly high-performing models, but still, they have a few weaknesses. One of them is that these models are prone to noise, which makes the image preprocessing an important task to be carried out on every input image. Therefore we also have preprocessed all the images and for that we adopted N4ITK algorithm [35], a bias correction technique which makes dataset images homogeneous. With further processing, the top 1% and bottom 1% intensities are abandoned as done in [7]. Finally, all the images are normalized with the mean value of 0 and the variance value of 1.

### 4.2. Proposed BrainSeg-Net

**Figure 2:**
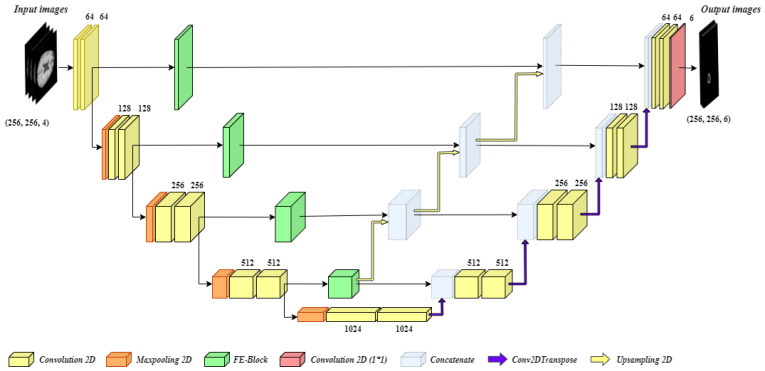
Architecture of the proposed BrainSeg-Net for MRI brain image segmentation.

The brain tumor segmentation is a complex task, and the biggest challenge in it to segment small scale tumors. Stronger contextual features are extracted at the deeper stage of the encoder; however, at this stage spatial and location, information is lost due to nonlinear transformations and continuous convolutions. By addressing this issue, an efficient model for brain image segmentation can be developed which can identify small-scale tumors as well. For this purpose, we have developed BrainSeg-Net which uses FE block which propagates spatial and location information during the process of upsampling (decoder side). Figure 2 shows the overall proposed architecture of BrainSeg-Net, and details regarding the BrainSeg-Net architecture are as follows.

As can be seen in Figure 2, the proposed BrainSeg-Net architecture is an encoder–decoder-based technique. The left side of the figure shows the encoder, which is a contracting path, while the right side of the figure shows the decoder, which is an expanding path. The input preprocessed image is of size 256×256 where there are four input channels. The encoder and decoder have four blocks with a single transition at bottom of the architecture. Every block of the encoder side consists of two convolution layers while three of the blocks also carry single max-pooling layer. The blocks of decoder consist of a dropout layer and two convolution layers, while the last decoder block contains an additional convolution layer with the filter size of 1×1. The transition block contains a max-pooling layer along with two convolution layers. The output of every encoder block is given to the next encoder block and the FE block. The output of the last block of the encoder is given to transition block while its output is given to the first decoder block. The sequence of decoder blocks starts from the bottom, while the output is taken from the last block of the decoder. The output of every decoder block undergoes Conv2DTranspose. A bridge connection between between every encoder block and its associated decoder block contains FE block. The output of the FE block is concatenated with the output of all the deeper stage FE blocks. Finally, the generated output is concatenated with the output of corresponding Conv2DTranspose layer of the decoder block and dropout is applied on the result, followed by convolution process carried out by two convolution layers of the decoder block.

The information collected by deeper FE blocks is upgraded and is shared with all available higher level decoder blocks. The upsampling operations performed in BrainSeg-Net are done using bilinear interpolation. The convolution layers used in BrainSeg-Net perform convolution with padding which allows achieving similar-sized output as of input. While the baseline architecture does not have this characteristic. The dropout ratio in BrainSeg-Net is kept at 0.3. All convolution layers of BrainSeg-Net contains Batch normalization and Rectified Linear Unit (ReLU) activation function excluding the last convolution layer which utilizes sigmoid activation function. The mathematical expression of ReLU and sigmoid activation function are given as

(1) $$ReLU\left(p\right)=\left\{\begin{array}{cc} 0, & \mathrm{for}\;p\leq 0\\ p, & \mathrm{for}\;p>0 \end{array}\right.$$

(2) $$Sigmoid\left(p\right)=\frac{1}{1+e^{-p}}$$

The BrainSeg-Net uses Adam optimizer along with the custom-designed loss function. The details regarding FE block and custom-designed loss function are discussed in following sub-sections.

#### 4.2.1. Feature Enhancer (FE)

The FE block helps to propagate the important location information along with spatial details. Sharing these features with all the higher level decoder blocks makes a significant improvement in segmentation performance. One of the most important contributions of the FE block is that it enhances the effective receptive field of the features maps. This increase in the effective receptive field will help in detecting larger regions by reading a more global feature hierarchy. While sharing important location details and spatial information, FE helps the architecture to identify small regions. Moreover, the skip connection of the FE block allows recovering the fine-grained details which got lost during the downsampling process. The architecture of the FE block is discussed as follows.

**Figure 3:**
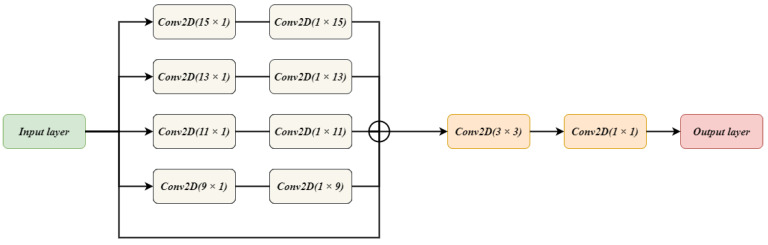
The Architecture of FE Block used in BrainSeg-Net.

Figure 3 illustrates the architecture of the FE block. The input from the encoder block is taken and given to five parallel connections. One connection acts as a skip connection, which helps to keep the spatial details from the encoder block unchanged. On the remaining four connections, two convolution layers are applied. A combination is made for the convolution layers. The first convolution layer has a filter size of N×1 while the second convolution layer has a filter size of 1×N. This combination is used rather than using a one convolution layer with N×N filter size. Such combination is used so that important features are extracted without losing location information. The experiments are carried out to observe the effect of using two cascaded convolutions rather than single convolution layer as discussed earlier. The cascaded system has depicted good performance when compared with the single convolution layer. The output of all the connections is summed together to get a single output. Two convolution layers with a filter size of 3×3 and 1×1, respectively, are applied on the attained output giving us the final result by FE block.

The FE block performs enhancement of the features given to it as an input. The FE block tries to extract features of features by keeping the minimal parameters. For keeping the parameters low, a special combination of convolution layers is applied. The block is not highly dense which helps in preserving the location information of the features.

#### 4.2.2. Custom-Designed Loss Function

The class imbalance is considered to be one of the biggest identified challenges in brain tumor segmentation. The difference between occupied regions between different classes can be understood in a better manner by considering Table 1. Table 1 illustrates the class distribution for BraTS 2017 dataset. In brain tumor MRI, an average occupied region by healthy tissue is 98.46%. Whereas the region occupied by edema, enhancing tumor, and non-enhancing tumor occupy 1.02%, 0.29%, and 0.23%, respectively. The large difference between region occupancy by different classes in brain tumor MRI has a tremendous effect on its segmentation accuracy.

To address the class imbalance issue, BrainSeg-Net adopts a custom-designed loss function where weight cross-entropy (WCE) and Dice Loss Coefficient (DLC) are summed up as a single loss function. The numerical representation of these loss functions is

(3) $$WCE=-\sum_{c}^{Q}w_{c}l_{c}lol\left(f_{c}\right)$$

(4) $$DLC=1-2\frac{\sum_{c}^{Q}w_{c}l_{c}f_{c}}{\sum_{c}^{Q}w_{c}\left(l_{c}+f_{c}\right)}$$

where Q is the total number of labels which in our case is 4 and${\;}^{\prime }c^{\prime }$ is the label. The $f_{c}$ represents the forecasted class of the pixel, $w_{c}$ is the allotted weight and $l_{c}$ is ground truth class of the pixel.

The total loss function can be represented as

(5) $$L_{total}=WCE+DLC$$

The loss function consists of two objective functions: WCE and DLC. Where DLC is responsible of predicting the segmented regions, while WCE performs classification of tissue cells.

## 5. Results and Discussion

For carrying out an extensive evaluation of the proposed model, we have done quantitative analysis as well as qualitative analysis. For the qualitative analysis of the proposed model, the evaluation metric we have used is Dice Score. The previous researches in literature have used dice score as a figure of merit so this will lead us to better performance comparison between state-of-the-art techniques and proposed technique. The Dice score is used to measure the similarity indexed between two sets suppose M & N which can be formulated as

(6) $$DiceScore=\frac{2\times |M\cap N|}{\left|M|+|N\right|}$$

where |M| and |N| are the cardinalities of sets M and N, respectively.

The proposed model is evaluated on three benchmark datasets: BraTS 2017, BraTS 2018, and BraTS 2019. The details of this dataset are discussed earlier in the paper. First of all, we evaluated the model on HGG cases of BraTS 2017. Total HGG cases were split into 80% and 20%, where the bigger chunk was used for training and a small chunk was used for testing. Table 2 shows the results obtained by BrainSeg-Net and its comparison with existing state-of-the-art techniques.

For HGG cases of BraTS 2017, the proposed model has exhibited a great increase in performance compared to the existing baseline and state-of-the-art techniques. The BrainSegNet attained a dice score of 0.903, 0.872, and 0.849 for the whole, core, and enhancing tumor, respectively. A segmentation improvement is observed for all the classes. BrainSegNet outperformed its baseline U-Net with high margin.

The second experiment is carried out by keeping the BraTS 2017 validation dataset as a test set and the whole BraTS 2017 training set is used to train the model. Two-hundred-and-eighty-five MR brain images are used to train the model and 57 MR scans are used to evaluate the performance. Table 3 represents the comparison of performance between BrainSeg-Net and existing models.

The experiment on BraTS 2017 validation dataset illuminates the high performance by BrainSeg-Net. The proposed model has improved the performance by 2.2%, 2.4%, and 2.9% for the whole, core, and enhancing tumor classes, respectively. When compared with the baseline architecture, BrainSeg-Net shows an improvement of 2.8% for the whole tumor, 3% for the core tumorm and 4.5% for the enhancing tumor. The large improvement in performance for enhancing tumor segmentation replicates that BrainSeg-Net is successfully able to resolve the problem regarding small-scale tumor segmentation.

The further experiment is carried out on BraTS 2018 validation dataset. The model is trained on 285 MRI scans of a training dataset of BraTS 2017, as BraTS 2018 do not have a separate training dataset. For the testing purposes, the BraTS 2018 validation dataset carrying 66 MR brain images is used. Table 4 illustrates the comparison between achieved results from BrainSeg-Net and the results achieved by existing techniques.

On the BraTS 2018 validation dataset, the BrainSeg-Net, respectively, achieves Dice scores of 0.773, 0.826, and 0.894 for enhancing tumor, core tumor, and whole tumor. The existing state-of-the-art results for this experiment are obtained by ResU-Net. The proposed model has successfully improved the performance for all the segmentation classes. The improvement for enhancing tumor is 0.5%, while for core tumor its 2.3 %. Both scenarios have small-scale regions that need to be segmented out. These results show that the information contribution of FE block at every deep layer has contributed towards the betterment of accurate segmentation.

To validate the generalization of the proposed model, we have evaluated it on the BraTS 2019 dataset. Where all training data of BraTS 2019 dataset are used to train the model and the validation data is used for testing. We have used 355 cases for the training purpose and 125 cases for the testing purpose. As this is the recent database, very limited results are reported by other works therefore we have compared our model with the baseline models. Table 5 shows the result comparison of BrainSeg-Net and baseline models.

The proposed BrainSeg-Net has achieved the highest performance for BraTS 2019 validation dataset. It has achieved the dice score of 0.869 for the whole tumor, 0.775 for the core tumor, and 0.708 for the enhanced tumor. BrainSeg-Net has outperformed its baseline models due to the effectiveness of the FE block which makes it easy to identify the small-scale tumor in brain tumor MRI.

**Figure 4:**
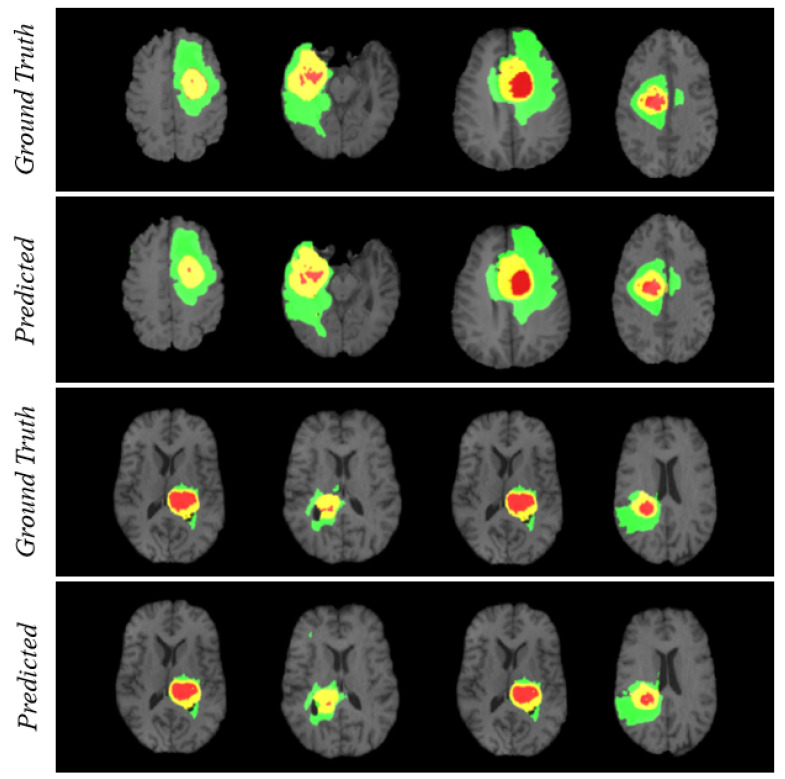
Visualization of segmentation performance by BrainSeg-Net architecture. Three colors in each case represents different classes. Red for necrosis and non-enhancing, green for edema, and yellow for enhancing tumor.

For visualizing the qualitative results, we have added Figure 4. The figure carries eight different cases where the region prediction by BrainSeg-Net is shown along with the labeled ground truth. By comparing both, we can have an idea about the reliability of the segmentation results achieved by BrainSeg-Net. The high resemblance between predicted and ground truth speaks about the high quality of BrainSeg-Net architecture.

## 6. Conclusions

The segmentation of MR brain tumor images is considered a complex task. Multiple neural network-based models are proposed in the literature for semantic segmentation of brain tumor MRI. Still, a gap of improvement exists. The biggest challenge is to segment the small-scale tumor as, due to the constant convolution operation and transformation in the neural network, an important location and spatial information get lost. Therefore, this paper proposed BrainSeg-Net, which addresses this issue and improves the semantic segmentation of MR brain tumor images. BrainSeg-Net uses FE block which, at every encoder stage, gets the feature map and performs the defined operation and tries to preserve the important information. The FE block converts the low-level features to middle-level features which minimize the information distortion during feature concatenation. Moreover, the FE block is responsible for improving the effective receptive field of architecture which again contributes towards architecture accuracy. The proposed model is evaluated on multiple benchmark databases. BrainSeg-Net has expressed a viable improvement in the results when compared with existing state-of-the-art semantic segmentation techniques for MR brain tumor images. The proposed BrainSeg-Net has also outperformed its baseline U-Net architecture. In future, we intend to improve this architecture further so that it can prove to be beneficial for human lives. The 2D U-Net has the restriction of important information loss in comparison to 3D U-Net. We have an intention to extend our research to explore 3D-based architecture doe improvement of segmentation performance.

## Figures and Tables

**Table 1:** Distribution of area occupancy by different classes in brain tumor MRI.

Class	Area Covered in %
Healthy Tissue	98.46
Edema	1.02
Enhancing Tumor	0.29
Non-Enhancing Tumor	0.23

**Table 2:** Results comparison on the BraTS 2017 HGG data.

Network	Whole	Core	Enhancing
CNN [36]	0.824	0.761	0.686
U-Net [23]	0.873	0.824	0.806
Densely CNN [37]	0.785	0.846	0.813
ResU-Net [38]	0.878	0.861	0.832
FCNN [39]	0.844	0.820	0.809
Proposed BrainSeg-Net	**0.903**	**0.872**	**0.849**

**Table 3:** Results comparison on the BraTS 2017 validation dataset.

Network	Whole	Core	Enhancing
U-Net [23]	0.870	0.762	0.700
ResU-Net [38]	0.873	0.768	0.716
PSPNet [26]	0.809	0.701	0.554
NovelNet [26]	0.876	0.763	0.642
Proposed BrainSeg-Net	**0.898**	**0.792**	**0.745**

**Table 4:** Results comparison on the BraTS 2018 validation dataset.

Network	Whole	Core	Enhancing
U-Net [23]	0.860	0.790	0.767
Ensemble Net [40]	0.881	0.777	0.773
MCC [41]	0.882	0.748	0.718
TTA [34]	0.873	0.783	0.754
ResU-Net [38]	0.887	0.803	0.768
Proposed BrainSeg-Net	**0.894**	**0.826**	**0.773**

**Table 5:** Results comparison on the BraTS 2019 validation dataset.

Network	Whole	Core	Enhancing
U-Net [23]	0.864	0.746	0.696
ResU-Net [38]	0.867	0.760	0.704
Proposed BrainSeg-Net	**0.869**	**0.775**	**0.708**

## Data Availability

Data sharing not applicable. No new data were created or analyzed in this study.
